# Supplementary material for: Arp2/3 complex-dependent actin remodeling is required for efficient RSV uncoating in A549 cells
Source: J Virol. 2026 Aug 31;100(9):e01117-26. doi: 10.1128/jvi.01117-26 (PMC13595987; doi:10.1128/jvi.01117-26)
Supplement: Supplementary file 1 [file jvi.01117-26-s0001.pdf]

Supplementary Information

Arp2/3 complex-dependent actin remodeling is required for efficient RSV uncoating in A549 cells

Enyu Xie [1], Maren Schubert [2], Jennifer Reeb [1], Benedikt Seligmann [3,4], Matthias Schaks [3,4], Mathias Müsken [5], Anika Steffen [6], Theresia E.B. Stradal [6], Sibylle Haid [7], Klemens Rottner [3,4,8], Christian Sieben [1,8,,9,§]

[1] Nanoscale Infection Biology Group, Helmholtz Centre for Infection Research, Inhoffenstraße 7, 38124 Braunschweig, Germany;

[2] Virus-like-particle Based Technologies Group, Helmholtz Centre for Infection Research, Inhoffenstraße 7, 38124 Braunschweig, Germany;

[3] Division of Molecular Cell Biology, Zoological Institute, Technische Universität Braunschweig, Spielmannstraße 7, 38106 Braunschweig, Germany;

[4] Molecular Cell Biology Group, Helmholtz Centre for Infection Research, Inhoffenstraße 7, 38124 Braunschweig, Germany;

[5] Central Facility for Microscopy, Helmholtz Centre for Infection Research, Inhoffenstraße 7, 38124 Braunschweig, Germany;

[6] Department of Cell Biology, Helmholtz Centre for Infection Research, Inhoffenstraße 7, 38124 Braunschweig, Germany;

[7] Institute for Experimental Virology, Centre for Experimental and Clinical Infection Research, a Joint Venture between the Helmholtz Centre for Infection Research and the Hannover Medical School, TWINCORE, Feodor-Lynen-Str. 7-9, 30625 Hannover, Germany;

[8] Braunschweig Integrated Centre of Systems Biology (BRICS), Braunschweig, Germany

[9] Institute for Genetics, Technische Universität Braunschweig, Spielmannstr. 7, 38106 Braunschweig, Germany;

[§] Correspondence should be addressed to christian.sieben@helmholtz-hzi.de

Supplemental materials

Table S1. Primers used in this study.

Target Gene	Forward (5′–3′)	Reverse (5′–3′))	Ref.
ACTR2 PCR primers	CAGCGAATTTGTCCATCCCAC	TAAGGTGCCAAAGAGACACTCA	
Sequencing primer	CATGAACCCTGGAAGTAGGC		
N	AGATCAACTTCTGTCATCCAGCAA	TTCTGCACATCATAATTAGGAGTATCAAT	[1]
NS1	CACAACAATGCCAGTGCTACAA	TTAGACCATTAGGTTGAGAGCAATGT	[2]
NS2	TTGATGAAAAACAGGCCACA	GGGAAAGTGCCATATTTTGTGT	[3]
HPRT1	GGGAGGCCATCACATTGTAG	AATCCAGCAGGTCAGCAAAG	[4]
IFNL1	GTTCAAATCTCTGTCACCAC	TTCAGCTTGAGTGACTCTTC	[5]
IFNB	TTGACATCCCTGAGGAGATTAAGC	TCCCACGTACTCCAACTTCCA	[5]

Abbreviations: Ref., reference; sgRNA, single-guide RNA; *N*, RSV nucleoprotein; *NS1*, RSV nonstructural protein 1; *NS2*, RSV nonstructural protein 2; *HPRT1*, hypoxanthine phosphoribosyltransferase 1; *IFNL1*, interferon lambda 1; *IFNB*, interferon beta 1.

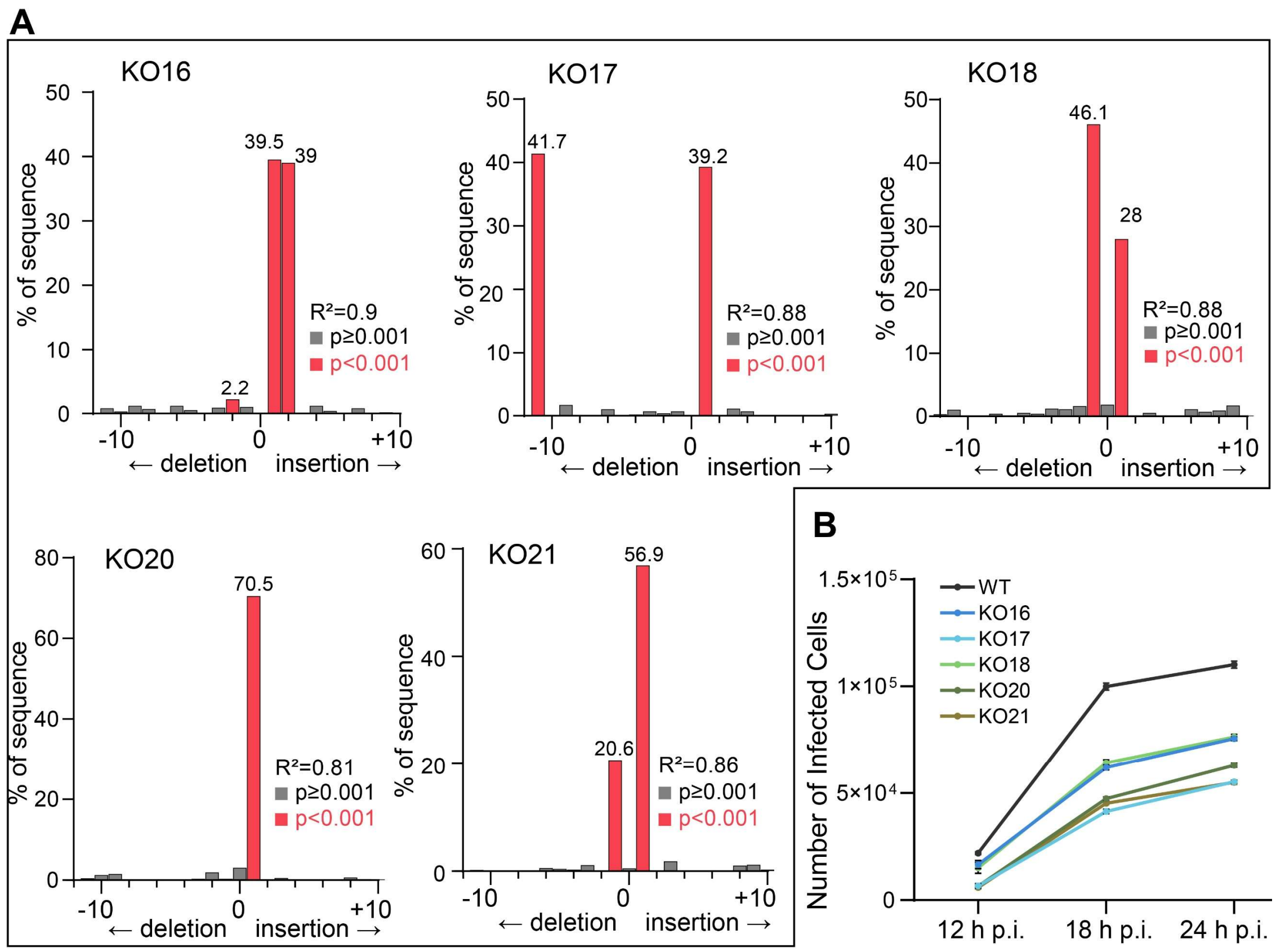

Fig. S1 | Arp2 knockout inhibits RSV infection in A549 cells at both early and late stages.

(A) TIDE sequence trace decomposition analysis of clonal cell lines derived from expanded clones, as indicated, upon A549 cell transfection with gene disruption construct PX459-Arp2. R^2 indicates goodness of fit.

(B) Time course of RSV-GFP infection (MOI = 3) in A549 WT and Arp2 KO cells quantified by live cell microscopy (Incucyte). Mean ± SD, n = three biological replicates.

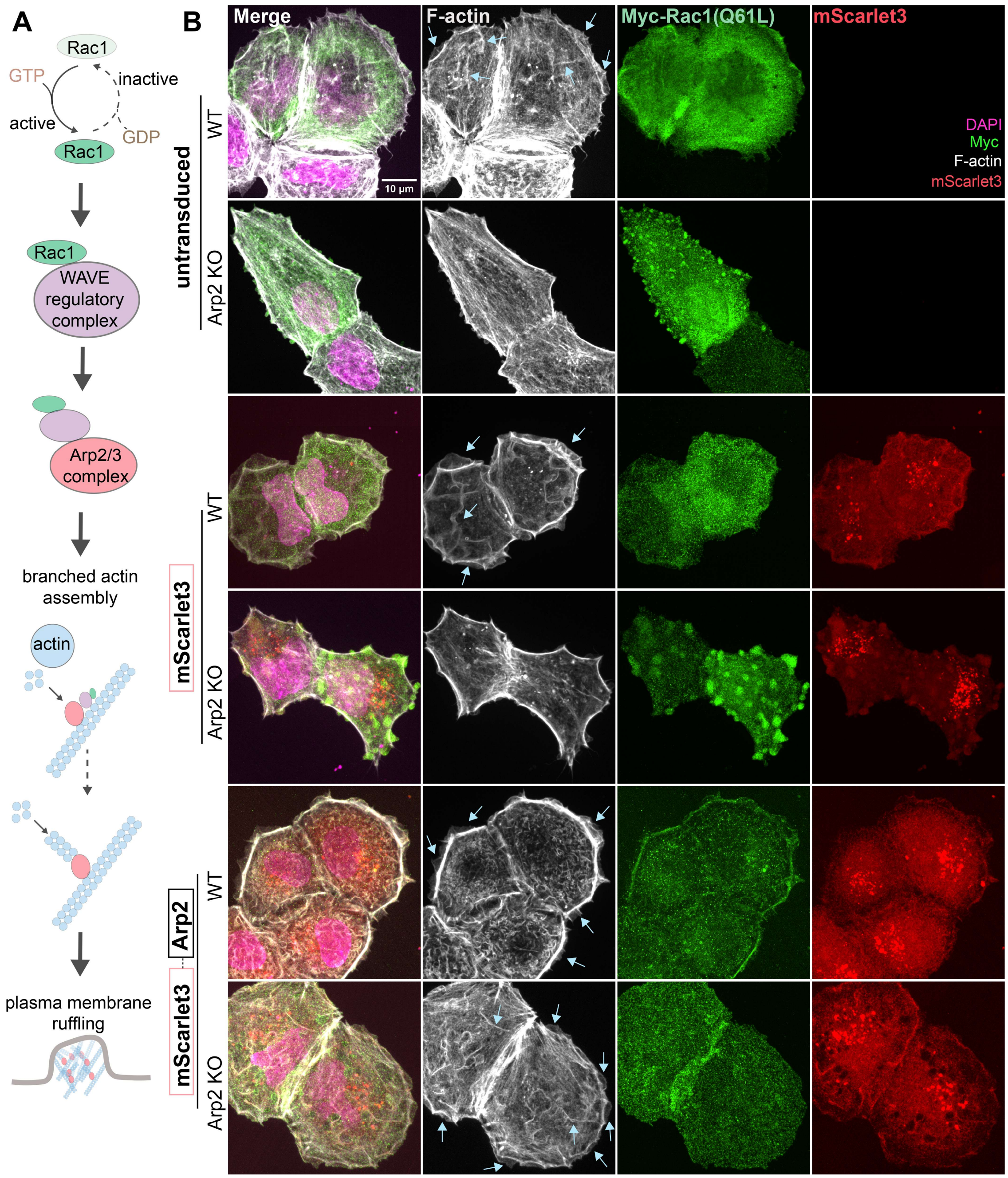

Fig. S2 | Arp2 rescue restores Rac1-induced membrane ruffling in Arp2 KO cells.

(A) Model of Rac1-induced membrane ruffling through Arp2/3 complex-dependent actin remodeling. Rac1 cycles between inactive GDP-bound and active GTP-bound states [6]. The constitutively active Rac1 (Q61L) mutant is defective in GTP hydrolysis and therefore mediates persistent Rac1 activation [7, 8]. Active Rac1 engages downstream effectors, specifically the WAVE regulatory complex, to activate the Arp2/3 complex in the lamellipodium [9-11]. Activated Arp2/3 complex promotes branched actin assembly, which drives local plasma membrane protrusion and membrane ruffling. Myc-Rac1(Q61L) was used to test the integrity of the Rac1-WAVE regulatory complex-Arp2/3 complex signaling axis in Arp2 knockout cells.

(B) Representative spinning-disk confocal microscopy images of cells overexpressing Myc-Rac1(Q61L). Cells were transfected with pRK5-Myc-Rac1(Q61L), fixed, permeabilized, and counterstained with anti-Myc antibodies (green), Alexa Fluor 647-conjugated phalloidin (pseudocolored gray), and Hoechst 33342 (pseudocolored magenta). Images are shown as maximum-intensity projections. Scale bar, 10 µm. Blue arrows point to representative lamellipodia and membrane ruffles.

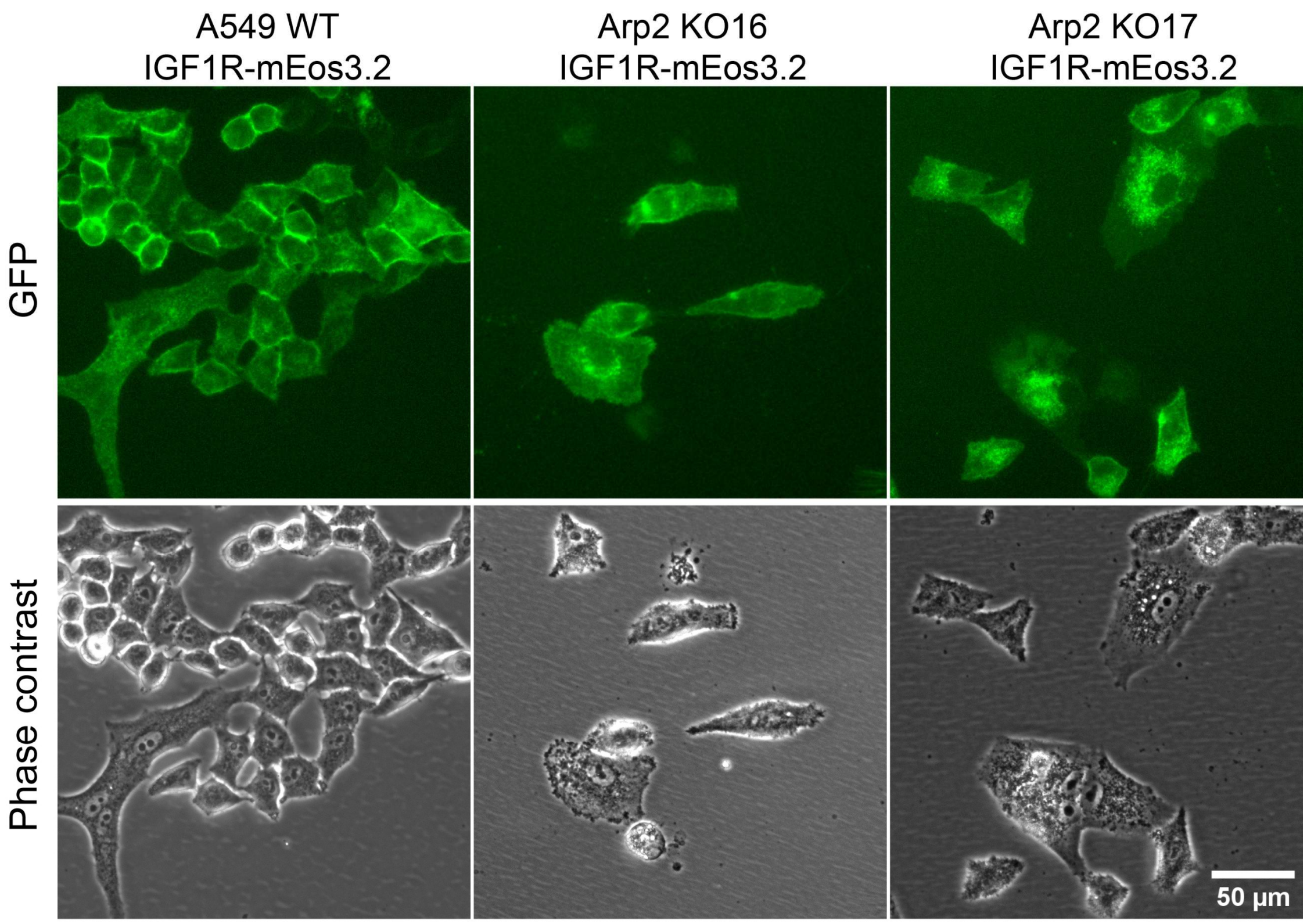

Fig. S3 | A549 cells stably expressing IGF1R-mEos3.2.

Representative images of WT and Arp2 KO A549 cell lines stably expressing IGF1R-mEos3.2.

Scale bar: 50 µm.

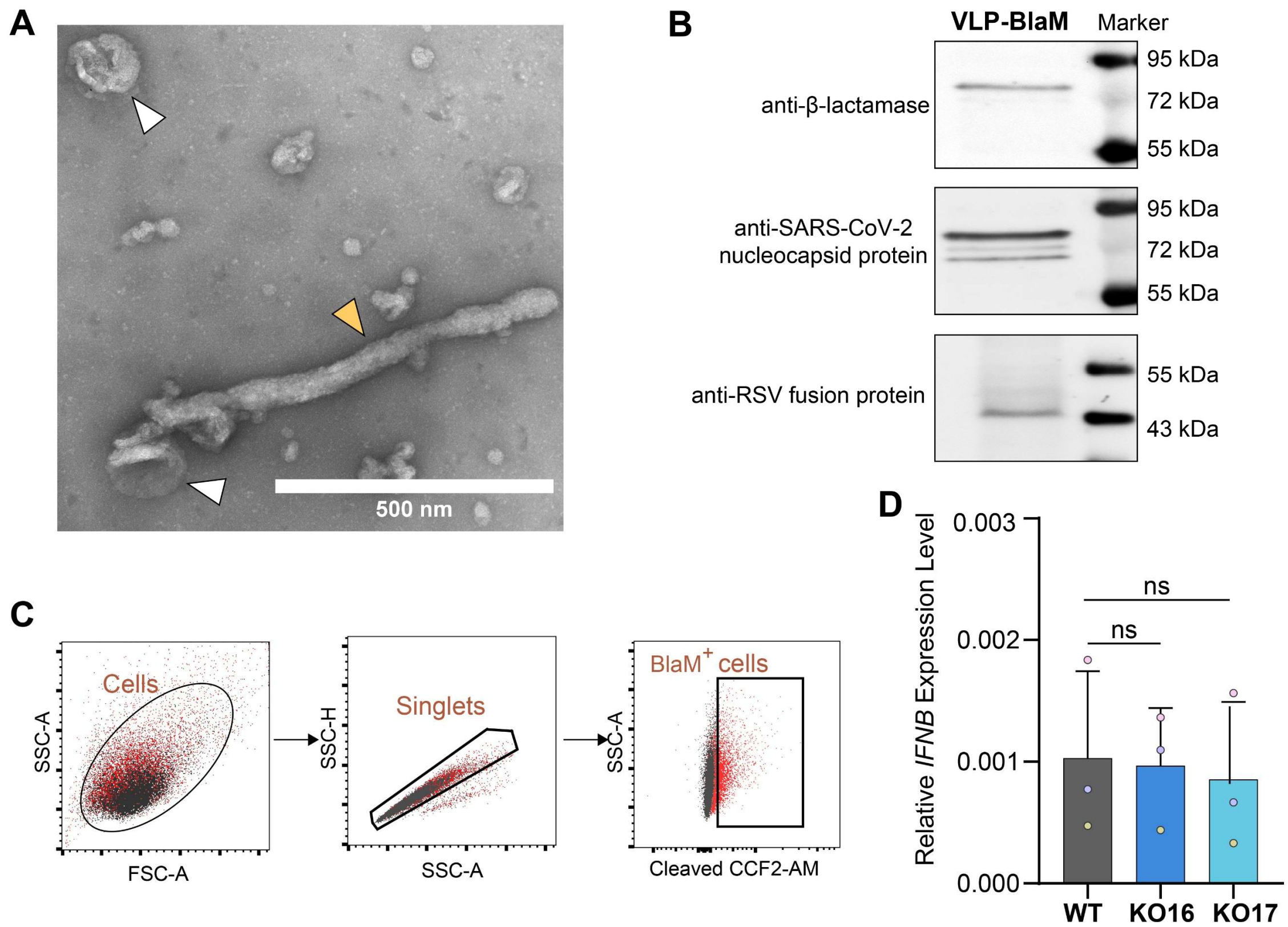

Fig. S4 | Validation of the uncoating assay and detection of *IFNB* expression level

(A) Representative transmission electron microscopy image of VLP-BlaM. White arrowhead indicates spherical VLP; yellow arrowhead indicates filamentous VLP.

(B) Western blot analysis of VLP-BlaM.

(C) Flow cytometry gating strategy for BlaM-positive cells. Gray dots, mock-infected controls; red dots, BlaM-VLP-infected cells. FSC-A, forward scatter-area; SSC-A, side scatter–area; SSC-H, side scatter-height.

(D) RT-qPCR analysis of *IFNB* expression level at 18 h post RSV-GFP infection. The transcripts were quantified and normalized to the cellular housekeeping gene *HPRT1* (hypoxanthine phosphoribosyltransferase 1) RNA levels. Combined data from three independent experiments (three biological replicates for each experiment). Each color represents one experiment; each dot is the experiment's mean. Mean ± SD. ns, not significant (two-way ANOVA).

References

1. van de Pol, A.C., et al., *Molecular quantification of respiratory syncytial virus in respiratory samples: reliable detection during the initial phase of infection.* J Clin Microbiol, 2010. **48**(10): p. 3569-74.
2. Huang, H., et al., *Impaired gamma delta T cell-derived IL-17A and inflammasome activation during early respiratory syncytial virus infection in infants.* Immunol Cell Biol, 2015. **93**(2): p. 126-35.
3. Yamamoto, K., et al., *Evaluation of consistency in quantification of gene copy number by real-time reverse transcription quantitative polymerase chain reaction and virus titer by plaque-forming assay for human respiratory syncytial virus.* Microbiol Immunol, 2018. **62**(2): p. 90-98.
4. Pinhu, L., et al., *Reference gene selection for real-time polymerase chain reaction in human lung cells subjected to cyclic mechanical strain.* Respirology, 2008. **13**(7): p. 990-9.
5. Hillyer, P., et al., *Expression profiles of human interferon-alpha and interferon-lambda subtypes are ligand- and cell-dependent.* Immunol Cell Biol, 2012. **90**(8): p. 774-83.
6. Bianchi-Smiraglia, A., et al., *Regulation of local GTP availability controls RAC1 activity and cell invasion.* Nature Communications, 2021. **12**(1): p. 6091.
7. Braga, V.M., et al., *The small GTPases Rho and Rac are required for the establishment of cadherin-dependent cell-cell contacts.* J Cell Biol, 1997. **137**(6): p. 1421-31.
8. Nobes, C.D. and A. Hall, *Rho GTPases Control Polarity, Protrusion, and Adhesion during Cell Movement.* Journal of Cell Biology, 1999. **144**(6): p. 1235-1244.
9. Steffen, A., et al., *Sra‐1 and Nap1 link Rac to actin assembly driving lamellipodia formation.* The EMBO Journal, 2004. **23**(4): p. 749-759.
10. Ridley, A.J., et al., *The small GTP-binding protein rac regulates growth factor-induced membrane ruffling.* Cell, 1992. **70**(3): p. 401-410.
11. Schaks, M., et al., *Distinct Interaction Sites of Rac GTPase with WAVE Regulatory Complex Have Non-redundant Functions in Vivo.* Curr Biol, 2018. **28**(22): p. 3674-3684.e6.
